# Evaluation of microfracture of traumatic chondral injuries to the knee in professional football and rugby players

**DOI:** 10.1186/1749-799X-4-13

**Published:** 2009-05-07

**Authors:** Masoud Riyami, Christer Rolf

**Affiliations:** 1 The Sheffield Centre of Sports Medicine, University of Sheffield, Sheffield, UK

## Abstract

**Background:**

Traumatic chondral lesions of the knee are common in football and rugby players. The diagnosis is often confirmed by arthroscopy which can be therapeutic by performing microfracture. Prospective information about the clinical results after microfracture is still limited.

**Aim:**

To evaluate the short-term outcome of microfractured lesions in professional football ad rugby players in terms of healing and ability to return to play.

**Methods:**

Twenty-four consecutive professional male players with isolated full-thickness articular cartilage defects on weight-bearing surface of femoral condyles were treated with microfracture. Clinical assessment of healing was done at three, six, 12 and at 18 months by using modified Cincinnati subjective and objective functional scoring. All 24 subjects were periodically scanned by 3-Tesla MRI on the day of the clinical evaluations and scored by the Henderson MRI classification for cartilage healing. A second look arthroscopy was carried out in 10 players five to seven months after surgery to evaluate lesion healing by using ICRS scoring system. This was done due to presence of discrepancy between a "normal" MRI and persistent clinical symptoms.

**Results:**

This study showed that 83.3% of players' resume full training between five to seven months (mean: 6.2) after microfracture of full-thickness chondral lesions of weight-bearing surface of the knee. Function and MRI knee scores of the 24 subjects gradually improved over 18 months, and showed good correlation in assessing healing after microfracture at six, 12 and 18 months (r^2 ^= 0.993, 0.986 and 0.993, respectively) however, the second look arthroscopy score proved to have stronger strength of association with function score than MRI score.

**Conclusion:**

We confirmed that microfracture is a safe and effective procedure in treating isolated traumatic chondral lesions of the load-bearing areas of the knee. Healing as defined by subjective symptoms and evaluated by MRI and a modified knee function score occurred between 5 to 7 months in most cases, which is a reasonable absence period for the majority of players to resume their normal sports activity without risking contracts and careers. MRI correlated well with the functional knee score, but neither of these methods were totally reliable in confirming healing at the defect site. Arthroscopic probing is therefore still the gold standard in our view. From a strict scientific stand point an untreated control group would be valuable to demonstrate that microfracture does not just mirror the natural course of healing.

## Introduction

Traumatic knee articular cartilage injuries are common findings during arthroscopy [1]. In a review study of 25,124 knee arthroscopies Widuchowski et al (2007) reported the incident of localized focal osteochondral or chondral lesions in 67 percent of patients of which 30 percent were isolated lesions [2].

These injuries present a therapeutic challenge, have little potential to heal, and have been identified as an important cause of permanent disability because of the high mechanical joint stress in athletes [3,4]. There are several choices the surgeons has in managing these articular surface defects, for example, arthroscopic microfracture [5-7], chondrocyte implantation [8], and osteochondral grafting [9], but what complicates the choice, however, is that only a few natural history studies show the long-term outcome of these procedures [3].

Microfracture is a technically simple and cost-effective treatment option for articular cartilage lesions of the knee [10]. This "marrow-based" strategy has produced a durable cartilaginous repair tissue when proper surgical techniques and postoperative rehabilitation protocols are followed [5]. Although several studies demonstrate the long-term efficacy of microfracture in elite athletes, as well as in traumatic chondral lesions [11,12], no investigation has focused on short-term functional outcome in professional footballers and rugby players in terms of lesion healing and their ability to return to play.

## Methods

**Figure 1:**
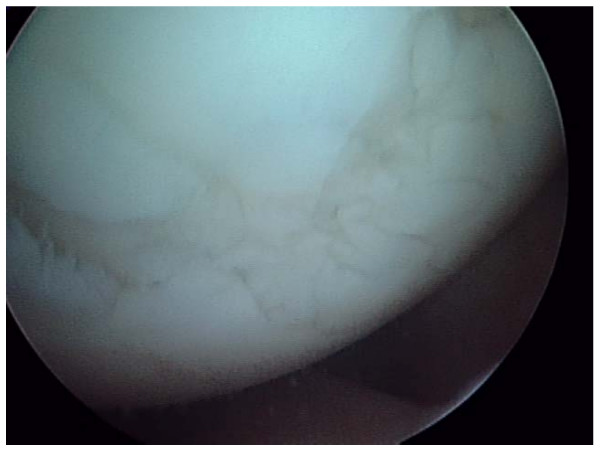
**A 22 year old professional football player with grade IV lesion on the medial femoral condyle**.

This is a prospective study of consecutive patients being either professional footballers or rugby players fulfilling criteria for microfracture treatment due to isolated chondral injuries to the knee. Ethical approval was granted by the Ethical Research Committee of the University of Sheffield. From October 2004 to December 2006 a total of 472 knee arthroscopies were performed at our centre of which most patients were professional or semi-professional football and rugby players. 42/472 was deemed to have isolated acute chondral lesion(s) on the weight-bearing surfaces of the femur or tibia. These subjects had isolated and well defined grade II–III or IV injuries (fig. 1). They all had an acute onset of knee pain and effusion as predominant symptoms, and the observed chondral defects were deemed to be the cause of the player's symptoms. Out of these 42 players 24 had full-thickness lesions were treated with microfracture, mean lesion size was 197 square mm (range: 63 to 275 square mm).

The preoperative duration of symptoms was two to three weeks. The indications and the decision to go ahead with this procedure were discussed with the player and Team medic's pre operatively, and confirmed in the operating theatre with the Team medic's attending the operation. The selected group consisted of full time professional (n = 15) and semi-professional (n = 9) players. The semi-professional players are those plays at lower division league and having part time jobs.

**Figure 2:**
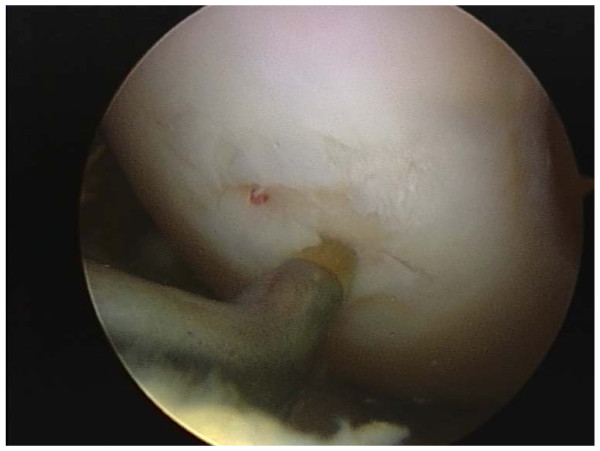
**grade IV lesion microfractured in a 26 year old rugby player**.

**Figure 3:**
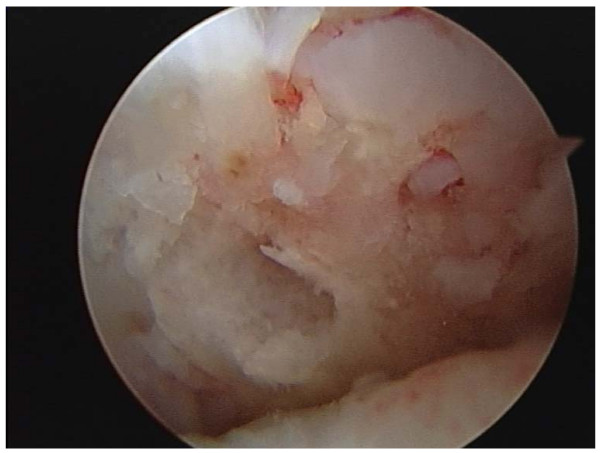
**Microfractured grade IV lesion in a 28 year old football player**.

All arthroscopies were performed under general anesthesia with standard techniques, using antero-medial, antero-lateral portals and tourniquet. During arthroscopy a systematic inspection of all joint components was undertaken. Prior to the microfracture, loose bodies were excised without removal of calcified cartilage and loose edges debrided, before the awl was used (fig. 2) to perforate the subchondral bone to a depth of 2 mm. A distance of 3 to 4 mm between the holes was left to preserve the integrity of the subchondral plate. The number of perforations for each lesion was decided according to the size of the lesion (fig. 3).

The rehabilitation protocol consisted of active motion which started immediately post-operatively, static quadriceps exercises and prone knee curls. Subjects were advised to use crutches and to be non weight-bearing for 6 weeks. This was followed by closed chain exercises guided by their team physiotherapist in close collaboration with the surgeon. Opened chain exercises were allowed after 3 to 6 months. Impact with pivoting such as running or jumping was not allowed until there were clear clinical (no effusion or tenderness on palpation) and radiological signs of healing.

Clinical assessment of healing was done at 3 months, 6 months, 12 months and at 18 months. The clinical assessment protocol involved modified Cincinnati subjective and objective functional scoring [13,14]. The function score was classified in comparison to uninjured knee performance as in Table 1. All 24 subjects were periodically scanned on the day of the clinical evaluations. This was done using a Philips Medical Systems (Best, Holland) 3-Tesla Intera Magnetic Resonance Scanner. An experienced musculoskeletal radiologist assessed the MR scans for signs of healing. To improve data collection and the processing of the MRI scores, the Henderson MRI classification for cartilage healing [15] was used. MRI score was classified in term of radiological healing as in table 2. Henderson classification was modified to suit this study. The signal intensity and the effusion scores were ignored as these are specific for autologouse chondrocytes implantation, but the same scale of one to four for defect fill and subchondral oedema was used, with one indicating the worst and four for the best result (table 2). Function and MRI scores at three month after the microfracture were considered as a base-line for the subsequent scores.

**Table 1:** Classification of the range of possible function scores.

Severely abnormal	41–50%
Abnormal	51–60%
	61–70%

Nearly normal	71–80%
	81–90%

Normal	91–100%

**Table 2:** MRI scoring guide

**Score**	**Defect filling**	**Sub chondral oedema**
0	As appear three months after micro fracture	As appear three months after micro fracture

1	Up to 25% more than the base-line	Less by up to 25%

2	> 25% to 50% from the base-line	Less by 26% – 50%

3	> 50% to 75% from the base-line	Less by 51% – 75%

4	> 75% to 100% filling	Less by more than 75%

No significant healing score 2–3

Incomplete healing score 4–5

Nearly complete healing score 6–7

Complete healing score 8

**Figure 4:**
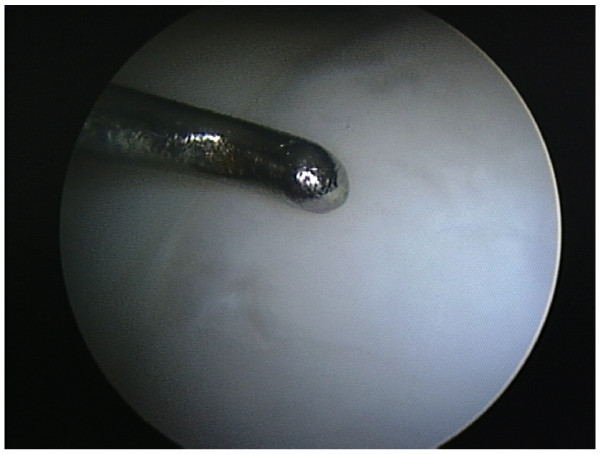
**Testing for firmness at second-look arthroscopy**.

Second-look arthroscopy was not initially planned for ethical and legal reasons. However, for 10 professional players in the premier league or just below, requests from the Team medics and managers for proof of lesion healing before allowing the players to resume play were set. Due to the low accuracy of standard MRI, arthroscopy was suggested and consented. This was of great value to this study, as the visual assessment and probing of the defect was considered to be the "gold standard" for the follow-up assessment. The second-look arthroscopy was done five to seven month (mean 5.8) from microfracture. The second-look arthroscopy allowed the visual assessment of the defect in term of quantitative and qualitative filling. The ICRS assessment form [1,16] for repair of cartilage was used to score the lesion site visually. The ICRS score was considered to be a guide only. The quality of the repaired tissue was assessed by probing the lesion for firmness (fig. 4). Should the lesion score high points by ICRS but feeling soft, the healing was considered to be incomplete and an additional period of rehabilitation was advised.

### Data and statistical analysis

Function and MRI scores were analyzed at 6 months, 12 months and 18 months. The healing progress by both modalities was determined, compared and correlated by the rank coefficient of correlation (r). The strength of the association between the two variables (r^2^) shows the probability that both modalities will give the same results. For 10 subjects these were compared with their ICRS scores.

## Results

**Figure 5:**
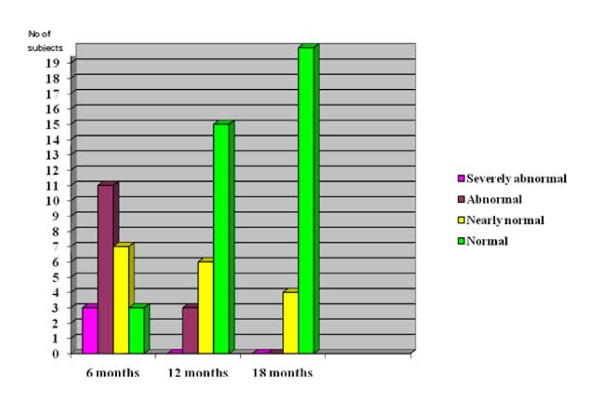
**Function score progress over time**.

This study shows that the function scores of the 24 subjects gradually improved over 18 months (fig. 5). At six months the function score for three (12.5%) subjects were "severely abnormal", 11 (45.8%) were "abnormal", seven (29.2%) were "nearly normal", and three (12.5%) were "normal". At 12 months there was no subjects with "severely abnormal" score, three (12.5%) were "abnormal", six (25%) were "nearly normal", and fifteen (62.5%) were "normal". At 18 months there were no subjects with "severely abnormal" or "abnormal" scores, four (16.7%) were "nearly normal", and 20 (83.3%) were "normal".

The pattern of figure 5 suggests that the period of maximum improvement of function score was between six to 12 months. The number of subjects, 12 (50%), with "normal" function score at 12 months was statistically significant (p < 0.05).

**Figure 6:**
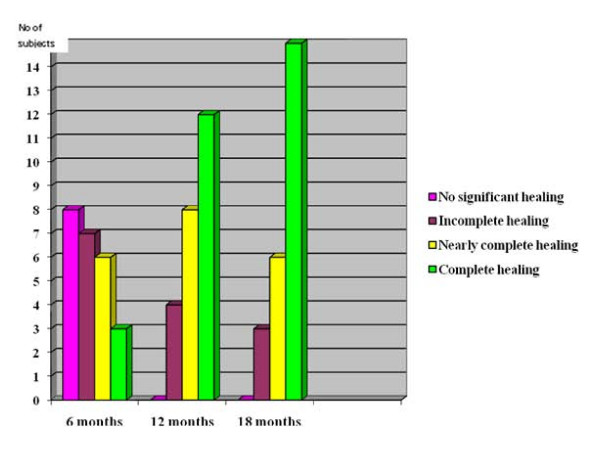
**MRI Score Progress**.

The MRI scores of the 24 subjects also gradually improved over 18 months (fig. 6). At six months the scores for eight (33.3%) subjects showed "no significant healing", seven (29.2%) showed "incomplete healing", six (25%) showed "nearly complete healing", and three (12.5%) showed "complete healing". At 12 months there were no subjects with a "no significant healing" score, four (16.7%) with an "incomplete healing" score, eight (33.3%) with a "nearly complete healing" score, and 12 (50%) with a "complete healing" score. At 18 months the scores of three (12.5%) subjects showed "incomplete healing", six (25%) showed "nearly complete healing", and 15 (62.5%) showed "complete healing".

Figure 6 also suggests that the period of maximum improvement of MRI score was between six to 12 months. The number of subjects, 12 (50%), who presented evidence of "complete healing" was statistically significant (p < 0.05).

The coefficient of rank correlation between function and MRI scores at six months was r = 0.996, which show a high strength of the association between these modalities of r^2 ^= 0.993. This means the improvement shown by one modality was nearly the same as that shown by the other. At 12 months the coefficient of rank correlation was r = 0.993, which also gives high strength of association between the modalities of r^2 ^= 0.986. At 18 months the coefficient of rank correlation was r = 0.996, with high strength of association between the modalities of r^2 ^= 0.993.

The second-look arthroscopy showed two out of 10 subjects scored ICRS "grade I". Their lesions were firm, and they were allowed to resume full training. Six subjects scored ICRS "grade II". Their lesions were soft on probing, and they were advised to continue closed chain exercises for a minimum of six weeks. The remaining two subjects scored ICRS "grade III", and were advised to have longer period of rehabilitation.

For the 10 subjects who underwent 2^nd ^look arthroscopy. The coefficient of rank correlation between arthroscopy and function scores was r = 0.958, which gives a strength of association of r^2 ^= 0.917. The coefficient of rank correlation between arthroscopy and MRI scores was r = 0.945, which gives the strength of association of r^2 ^= 0.894.

By considering arthroscopy as the "gold standard" for healing, this indicates that the function score had a higher strength of association with the arthroscopy score than the MRI score.

Nine players resumed their full training at six month despite the fact that functional assessment and MRI images revealed incomplete healing in the majority of subjects at this period, eleven resumed in the second 6-month period, and four resumed in the third 6-month period (table 3).

**Table 3:** Number of players resumed full training over the 18 months

**Period of resuming play**	**No. of players**
1^st ^6-month	9

2^nd ^6-month	11

3^rd ^6-month	4

**Total**	**24**

The subsequent function scores of the nine players that resumed full training at 6 months showed gradual improvement, they achieved the normal score by 18 months however, the MRI of only five showed complete healing, three nearly complete healing and one incomplete healing.

## Discussion

This study showed satisfactory functional and MR images outcome of microfracture in all 24 studied players. They were all back to play within 18 months despite the fact that the MR images of only 15 players showed "complete healing" scores. The remaining nine had less MRI scores. The decision of allowing subjects to resume full training was taken when clinical examination showed clear evidence of lesion healing, with absence of symptoms, effusion, tenderness, and with a negative compression/rotation test. Therefore, these nine subjects were considered to have the same level of healing as the rest of the subjects. The MR images of these nine subjects showed satisfactory defect filling but with persistence of a subchondral oedema-like signal that lowered their scores. This phenomenon is common after all types of chondral lesion repair [1,17]. The subjects in this study were competitive athletes, this provides a significant selection bias for the return to play since it has been shown that their return rate for high level athletes is better [18] and those professional athletes may have a higher rate of return since their motivation is much higher than that for recreational athletes. The return rate of this study can not be extrapolated to recreational athletes, since the personnel and facilities for rehabilitation available to professional players are much better than for the average recreational athlete. In comparison with other repair techniques, the return to play period for microfracture is shorter than for subjects received autologous cartilage transplantation as it was reported by Mithoefer et al. that 87% players maintained their ability to play soccer 52 +/- 8 months postoperatively [19]. A comparison study on 57 young athletes by Gudas et al. showed that 93% of athletes who received autologous osteochondral transplantation and 52% of the athletes who received microfracture returned to sports activities at the pre-injury level at an average of 6.5 months [20].

This study showed that microfracture produced durable repair tissue in short-term but for how long? Several studies demonstrated the long-term efficacy of microfracture in elite athletes, as well as in traumatic chondral lesions for subjects less than 40 years [3,4]. Other studies showed that microfracture has good short-term result in the treatment of small cartilage defects and a deterioration in function score starts 18 months after surgery, and the best prognostic factors have young patients with defects on the femoral condyles [12]. The subjects in this study were young with small lesions on femoral condyles which are factors favor them to have long-term repair durability.

Early evidence of lesion healing was seen on MRI as early as three months. By six months 50% of subjects had MRI evidence of complete healing whilst their function scores were normal. Progress of healing as shown by MRI was not always associated with the same degree of functional progress, and vice versa. However, overall the progress shown by MRI and function scores were highly comparable (r^2 ^= 0.993) for the whole period. This study also showed that lesion healing after microfracture is between six to12 months for the majority of subjects. Twenty (83.3%) of the subjects resumed full training and games in this period.

The results revealed a high correlation between MR images and function scores. At six months the strength of association was 99 percent (r^2 ^= 0.993). With progression of healing the strength of association at 12 months was slightly lower 98.6 percent (r^2 ^= 0.986). The strength of association between MR images and function scores at 18 months was again 99 percent (r^2 ^= 0.993). These two non-invasive modalities would be ideal for monitoring healing in daily clinical practice if they proved to be reliable and valid in comparison to the macroscopic healing. The correlation between defect fill shown by MRI and function score at 36 month was reported as 0.84 by Kreuz et al [12]. Mithoefer et al. found that all knee with good fill demonstrated improved knee function and poor fill grade is associated with limited short-term durability [19].

Brittberg and Winalski [1] in their evaluation of cartilage injuries and repair found that the subchondral oedema-like signal regresses as the repair site heals, but the precise timeline for the normalization of the marrow signal is unknown. This study showed nine (37.5%) subjects had a persistent subchondral oedema-like signal which extended beyond the period of the study. In a long-term follow-up of microfracture at 36 months, Kreuz et al [12] also found persistence of marrow oedema in some patients.

The second look arthroscopy was regarded the gold standard for assessing lesion healing in this study, where function scores provided 92 percent (r^2 ^= 0.917) of the information provided by arthroscopy scores, whilst MRI scores provided 89 percent (r^2 ^= 0.894) of the information provided by arthroscopy scores. Both function and MRI scores are indirect assessments of healing. The functional score reflects a subject's condition and MRI provides images for reading. Arthroscopy scores on the other hand, provide direct real time assessments. It is possibly unethical to subject every repaired case to arthroscopy to assess healing so it is promising that both non-invasive modalities showed to provide acceptable alternatives for assessing healing.

It must be stressed that there are no studies comparing microfracture with natural healing and we did not have such control group. Little is known about the natural course of chondral defects, particularly if and when they give clinical symptoms or radiographic signs of deterioration of the knee joint. Therefore, it is not known if any of the treatments that have been recommended for isolated chondral defects alter the natural course of the untreated lesion. No controlled studies have been done to determine whether treatment provides improvement over the natural history of the injury. Thus, scientifically, it is difficult to make a good decision regarding when, or even if, to treat these defects. Subjects with chondral lesions may have periods of time when they are symptomatic followed by times when they can be active without symptoms. The subjects in this study were symptomatic to the extent that they could not perform in their highly demanding sport, and we do believe that microfracture should be regarded as an appropriate treatment option.

In a study by Shelbourne et al [21] of the outcome of untreated traumatic articular cartilage defects of the knee, they followed 125 anterior cruciate ligament reconstructed patients who had associated chondral defects noticed at the time of reconstruction. They found that the outcome shown by the IKDC score at ten years was similar to that for the control group of ACL reconstructed patients without chondral defects. However, they have not suggested that there are no articular cartilage defects that will benefit from an articular cartilage restoration procedure.

Few studies in the past have discussed the outcome of microfracture by using function and MRI scores simultaneously. Those that did were in mosaicplasty as in the study of Gudas et al [20,22]. The design and criteria of assessment of those studies were different from this study. Therefore, these made the results we have achieved difficult to assess and compare.

## Conclusion

Microfracture is a safe and effective procedure for treatment of full-thickness isolated traumatic chondral lesions of the load-bearing areas of the knee in athletes. Significant defect healing and satisfactory clinical function out-come occurred between 5 to 7 months in most cases, which is a reasonable absence for the majority of subjects to resume their normal sports activity without risking contracts and careers. From a strict scientific stand point an untreated control group would be valuable for showing that microfracture does not just mirror the natural course of healing. MRI shows a high correlation with the clinical functional out-come in assessing lesion healing, but neither of these methods are totally reliable in confirming healing at the defect site and arthroscopy is therefore still the gold standard in our view.

## Competing interests

The authors declare that they have no competing interests.

## Authors' contributions

MR designs the study, collected data, did all the analysis, and draft the manuscript. CR participated in study design, operated on patients, and contributed in writing the manuscript. Both authors read and approved the final manuscript

## References

[B1] Brittberg M, Winalski CS (2003). Evaluation of cartilage injuries and repair. J Bone Joint Surg Am.

[B2] Widuchowiski W, Widuchowiski J, Trazaska T (2007). Articular cartilage defects: study of 25,124 knee arthroscopies. Knee J.

[B3] Johnson D (2003). Articular cartilage intervention evolve CME. Am Acad Orthop Surg.

[B4] Mithoefer K, Williams RJ, Warren RF, Wickiewicz TL, Marx RG (2006). High-impact athletics after knee articular cartilage repair: A prospective evaluation of the microfracture technique. Am J Sports Med.

[B5] Steadman JR, Rodkey WG, Rodrigo JJ (2001). Microfracture: Surgical technique and rehabilitation to treat chondral defects. J Clin Orthop.

[B6] Steadman JR, Rodkey WG, Briggs KK (2002). Microfracture to treat full-thickness chondral defects: Surgical technique, rehabilitation and outcomes. J Knee Surg.

[B7] Mithoefer K, Williams RJ, Warren RF, Potter HG, Spock CR, Jones EC, Wickiewicz TL, Marx RG (2005). The microfracture technique for the treatment of articular cartilage lesions in the knee. A prospective cohort study. J Bone Joint Surg Am.

[B8] Brittberg M, Lindahl A, Nilson A, Ohlsson L, Isaksson O, Peterson L (1994). Treatment of deep cartilage defects in the knee with autologous chondrocyte transplantation. N Engl J Med.

[B9] Bugbee W, Emmerson BC, Jamali AA (2003). Fresh osteochondral allografting in the treatment of osteochondritis dissecans of the femoral condyle. AAOS 70th annual meeting.

[B10] Steadman JR, Miller BS, Karas SG (2003). Patient satisfaction and outcome after microfracture of the degenerative knee. J Knee Surg.

[B11] Steadman JR, Briggs KK, Rodrigo JJ, Gill TJ, Rodkey WG (2003). Outcome of microfracture for traumatic chondral defects of the knee: average: 11-years follow-up. J Arthroscopy.

[B12] Kreuz PC, Erggelet C, Steinwachs MR, Krause SJ, Lahm A, Niemeyer P, Ghanem N, Uhl M, Sudkamp N (2006). Is microfracture of chondral defects in the knee associated with different results in patients aged 40 years or younger?. J Arthro Relt Surg.

[B13] Barber SD, Noyes FR, Mangine RE, McCloskey JW, Hartman W (1990). Quantitative assessment of functional limitation in normal and anterior cruciate Ligament deficient knee. Clinical orthopaedic and related research.

[B14] Barber-Westin SD, Noyes FR, McCloskey JW (1999). Rigorous statistical reliability, validity, and responsiveness testing of the Cincinnati knee rating system in 350 subjects with uninjured, injured, or anterior cruciate ligament reconstructed knees. Am J sports Med.

[B15] Henderson I, Tuy B, Connell D, Oakes B, Hettwer W (2003). Prospective clinical study of autologous chondrocyte implantation and correlation with MRI at three and 12 months. J Bone Joint Surg (Br).

[B16] (2004). ICRS cartilage evaluation package. http://www.Cartilage.com.

[B17] Winalski CS, Minas T (2000). Evaluation of chondral injuries by magnetic resonance imaging: repair assessment. Op Tech Sports Med.

[B18] Blevins FT, Steadman JR, Rodrigo JJ, Silliman J (1998). Treatment of articular cartilage defects in athletes: An analysis of function outcome and lesion appearance. Orthopedics.

[B19] Mithofer K, Peterson L, Mandelbaum B, Minas T (2005). Articular cartilage repair in soccer players with autologous chondrocyte transplantation: functional outcome and return to competition. Am J Sports Med.

[B20] Gudas R, Stankevicius E, Monastyreckiene E, Pranys D, Kalesinskas RJ (2006). Osteochondral autologous transplantation versus microfracture for the treatment of articular cartilage defects in the knee joint in athletes. J Knee Surg Sports Traumatol Arthrosc.

[B21] Shelbourne KD, Jari S, Gray T (2003). Outcome of untreated traumatic articular defects of the knee: A natural history study. J Bone Joint Surg (Am).

[B22] Gudas R, Kalesinskas RJ, Monastyreckiene E, Valancite A, Trumpickas V (2003). Osteochondral transplantation in the treatment of knee joint cartilage defects. Medicina (Kaunas).

